# Association of arterial stiffness with aortic calcification and tortuosity

**DOI:** 10.1097/MD.0000000000016802

**Published:** 2019-08-16

**Authors:** Inki Moon, Kwang Nam Jin, Hack-Lyoung Kim, Hyeon Jeong Suh, Woo-Hyun Lim, Jae-Bin Seo, Sang-Hyun Kim, Joo-Hee Zo, Myung-A Kim

**Affiliations:** a Department of Internal Medicine; b Department of Radiology, Boramae Medical Center, Seoul National University College of Medicine, Seoul, Republic of Korea.

**Keywords:** aortic calcification, aortic tortuosity, arterial stiffness, brachial-ankle pulse wave velocity

## Abstract

Impact of arterial stiffness on aortic morphology has not been well evaluated. We sought to investigate the association of brachial-ankle pulse wave velocity (baPWV) with aortic calcification and tortuosity.

A total of 181 patients (65.4 ± 10.4 years, males 59.7%) who underwent computed tomographic angiography and baPWV measurement within 1 month of study entry were retrospectively reviewed. Aortic calcification was quantified by the calcium scoring software system. Aortic tortuosity was defined as the length of the midline in the aorta divided by the length of linear line from the aortic root to the distal end of the thoraco-abdominal aorta. In simple correlation analyses, baPWV was correlated with aortic calcification (*r* = 0.36, *P* < .001) and tortuosity (*r* = 0.16, *P* = .030). However, these significances disappeared after controlling for confounders in multivariate analyses. Factors showing an independent association with aortic calcification were age (*β* = 0.37, *P* < .001), hypertension (*β* = 0.19, *P* = .003), diabetes mellitus (*β* = 0.12, *P* = .045), smoking (*β* = 0.17, *P* = .016), and estimated glomerular filtration rate (*β* = –0.25, *P* = .002). Factors showing an independent association with aortic tortuosity were age (*β* = 0.34, *P* < .001), body mass index (*β* = –0.19, *P* = .018), and diabetes mellitus (*β* = –0.21, *P* = .003).

In conclusion, baPWV reflecting arterial stiffness was not associated with aortic calcification and tortuosity. Traditional cardiovascular risk factors were more influential to aortic geometry. Further studies with a larger sample size are needed to confirm our results.

## 1 Introduction

Arterial stiffness represents adverse structural and functional changes in the vessel wall. Aging as well as mechanical and chemical stresses, such as arteriosclerosis, blood pressure, and chronic inflammation, are involved in loss of compliance that processes through loss of elastic fibers, increased collagen deposition, and vessel wall calcification, leading to arterial stiffening.^[1,2]^ In clinical practice, information on arterial stiffness is useful and important, because it has been suggested that the measurement of arterial stiffness is an independent predictor of cardiovascular mortality and morbidity.^[3,4,5]^

Pulse wave velocity (PWV) is the most widely used tool to measure arterial stiffness.^[6,7]^ Although carotid-femoral PWV (cfPWV) has been considered a gold standard, the cfPWV measurement requires technical skill and causes inconvenience to patients during femoral artery palpation.^[1]^ As a more recently introduced method for arterial stiffness, brachial-ankle PWV (baPWV) is easy, simple, and timesaving to measure.^[8]^ In addition, as the prognostic value of baPWV has been proven in previous clinical studies and meta-analyses.^[9,10,11]^ baPWV may be a reliable tool to measure arterial stiffness.

The aorta should provide not only tunnels for blood flows but also vascular buffering to each of the left ventricular contractions, for its distensibility and stiffness were very important in our circulatory system. Distensibility and stiffness are determined by the diameter, wall thickness, and histological structures of vessels, such as smooth muscle cells, elastin, collagen, and fibrin fibers.^[12]^ Vascular calcification is one of the mechanisms that develop arterial stiffness.^[1,13]^ With recent advances in multidetector computed tomography (MDCT), aortic calcification has been quantified using computed tomography (CT) to evaluate atherosclerosis and cardiovascular risk.^[14]^ Besides aortic calcification, aortic tortuosity can be measured simultaneously using computed tomographic angiography (CTA) datasets. Recent studies show the usefulness of measuring aortic tortuosity for the evaluation of aortic aneurysm prior to endovascular repair.^[15,16]^ Although several studies have reported that aortic calcification had a significant correlation with arterial stiffness,^[17,18,19,20,21]^ there has been a lack of data on the association between arterial stiffness and aortic tortuosity. This study aimed to evaluate the association of baPWV with the calcification and tortuosity of the aorta assessed by CTA.

## 2 Materials and methods

### 2.1 Study population

This study was performed in a single center. Between January 2010 and January 2016, a total of 241 subjects who underwent both baPWV measurement and thoracoabdominal CTA within one month of study entry were retrospectively included. CTA was performed to evaluate cardiac symptoms such as chest pain, to preoperatively examine patients for cardiac surgery, including valve replacement, coronary artery bypass graft surgery, and to detect aortic aneurysm. In addition, the baPWV measurement was performed to evaluate atherosclerotic burden on subjects, which was determined by the attending physician. We exclude 60 patients with the following conditions: aortic aneurysm (maximal aortic diameter >40 mm) (n = 15) and abnormal ankle-brachial index (<0.9 or ≥1.4) (n = 45). Finally, 181 subjects were included in this study. Approval of the study protocol was obtained from the institutional review board of Boramae Medical Center (Seoul, Republic of Korea; 16–2016-23). Written informed consent was waived by the institutional review board because of the retrospectively designed study and routine nature of the information.

### 2.2 Study data

Body mass index (BMI) was calculated by dividing weight in kilograms by the squared of height in meters (kg/m^2^). Data on underlying medical conditions, including hypertension, diabetes mellitus, hyperlipidemia, and smoking, were acquired. Hypertension, diabetes mellitus, and dyslipidemia were defined based on the previous diagnosis or current medications for controlling them. Smoking was defined as a history of smoking during the last year. Coronary artery disease included previous myocardial infarction or coronary revascularization. Venous blood for laboratory study was sampled after more than 8 hours of fasting. The white blood cell count, hemoglobin, creatinine, total cholesterol, low-density lipoprotein cholesterol, high-density lipoprotein cholesterol, triglyceride, and C-reactive protein were measured. Patient with chronic kidney disease was defined as who had an estimated glomerular filtration rate (eGFR) below 60 mL/min/1.73 m^2^. The eGFR was calculated by the following formula: 175 × serum creatinine^–1.154^ × age^–0.203^(× 0.742 if a woman). Left ventricular ejection fraction was calculated by Simpson's biplane method in transthoracic echocardiography. Information on cardiovascular medication was collected by reviewing the subject's medical records.

### 2.3 baPWV measurement

The baPWV was measured by the volume-plethysmography (VP-1000; Colinc Co. Ltd., Komaki, Japan) according to the manufacturer's recommendations.^[10,22]^ Patients were examined in the supine position after more than 5 minutes of rest. Caffeine consumption and cigarette smoking were forbidden, and regular medications were allowed before the examination. The cuffs were mounted on both upper arms and ankles. The blood pressure, heart rates, pulse volume waveform, and phonogram were measured simultaneously. We measured baPWV by estimating the difference of pulse wave time between the brachial and posterior tibial arteries. The mean value of left and right baPWV was analyzed and used in the analysis. These measurements were made by one experienced operator who was blind to information on patients. The coefficient of variation in baPWV measurement for intra-observer reliability was 5.1% in our laboratory.^[22]^

### 2.4 CT acquisition and post-processing

Thoraco-abdominal CT angiography (n = 174) or thoracic CT angiography (n = 7) were performed with a 16-channel MDCT scanner (Light-Speed; GE Healthcare, Little Chalfont Bucks, UK) (n = 160) or a 64-channel MDCT scammer (Brilliance; Philips Medical Systems, Cleveland, OH, USA) (n = 9) or a 128-channel MDCT scanner (Brilliance or Ingenuity; Philips Medical Systems) (n = 12). The sequence for performing CT scans includes both non-contrast media enhancement and contrast-enhanced scans in the cranio-caudal direction. Thoraco-abdominal or thoracic CTA was evaluated from the bulb level of the carotid to the kidney mid-pole level or inguinal area. The 120 to 140 mL bolus of Iopamidol (Iopamiro 300; Bracco, Milan, Italy) were injected intravenously, followed by flushing with 20 mL of saline (3.0 mL/s); contrast-enhanced scans were achieved. Helical scan data were achieved by collimation of 64 × 0.625 mm, 16 × 1.5 mm, or 64 × 0.6 mm, with a 0.42 or 0.5 seconds of the rotation speed, a 1.11 to 1.25 of pitch, and 120 kilovolts (peak). The effective milliampere-seconds set by the automatic tube current modulation technique were and between 120 and 200. We reconstructed transverse datasets in 1.2 mm or 1.5 mm thickness in increments of 1.2 mm or 1.5 mm. The image results were wired to the workstation (iSP, Philips Medical Systems) for analysis.

### 2.5 Quantification of aortic calcification and measurement of aortic tortuosity

**Figure 1:**
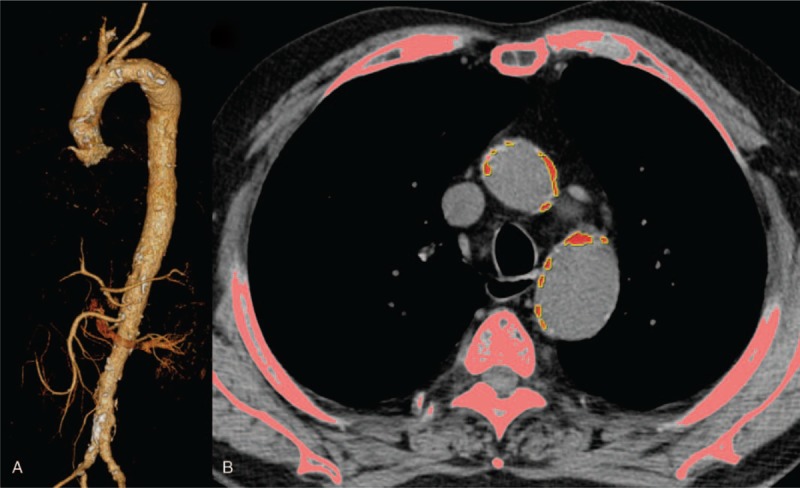
Measurement of aortic calcifications. Three-dimensional volume rendering image of thoracoabdominal CT angiography shows multiple atherosclerotic wall calcifications in the aorta, which are depicted as white patches. (A) Post-processing in the dedicated work stations enables the automated detection of bone and aortic calcifications. Semi-automated identification of aortic calcification was necessary for defining the aortic calcifications to differentiate the aortic calcium from the bone or calcified lymph nodes. Axial image obtained at the level of aortopulmonary window shows the red-colored aortic calcifications. (B) The total amount of aortic calcification measured 30,539.

**Figure 2:**
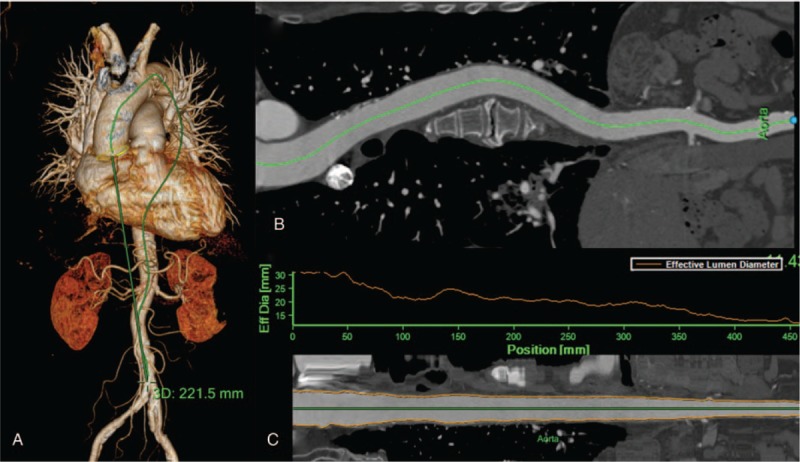
Measurement of aortic tortuosity. Three-dimensional volume rendering image of thoracoabdominal CT angiography shows that the distance between the aortic root and the aortic bifurcation is 221.5 mm. (A) Curved multiplarnar reconstruction image shows the centerline of the whole aortic lumen from the aortic root to the aortic bifurcation. (B) Straightened multiplanar reconstruction image shows that the length of the center line is 473.2 mm. Aortic tortuosity was estimated at 0.47 (= 221.5/473.2).

Non-contrast enhanced axial CT images were examined by an experienced cardiothoracic radiologist using the workstation. The calcium scoring software system was used to quantify aortic calcification, and the threshold was 130 Hounsfield units.^[23]^ The calcification was semi-automatically read by the dedicated radiologist’ on each axial CT image (Fig. 1). After scanning the whole thoracic aorta, the calcification was scored in the workstation. The wall calcifications in the thoracic aorta were detected from the aortic root, which is just above the left main coronary artery, to the level of the diaphragmatic crura, which is the bottom of both ventricles. The calcifications within the subclavian or carotid arteries were not read, and the abdominal aortic calcifications were not measured, because it is difficult to differentiate between the calcification and the adjacent vertebrae on the dedicated software. Using dedicated software, the thoracoabdominal aorta and its midline were automatically extracted from the aortic root and the iliac bifurcation. Tortuosity of the aorta, which was defined as the length of the midline in the aorta divided by the length of linear line from the aortic root to the distal end of the thoracoabdominal aorta, was calculated (Fig. 2). The distal ends were the iliac bifurcation for the thoracoabdominal aorta and the thoracoabdominal aorta junction for the thoracic aorta.

### 2.6 Statistical analysis

Consecutive variables are expressed as mean ± standard deviation, and categorical variables are expressed as percentages. We compared continuous variables between groups using Student's *t* test, and categorical variables using the chi-square test. Simple and multivariable linear regression analyses were performed to investigate whether baPWV is an independent factor for aortic calcification and tortuosity by adjusting for age, sex, BMI, hypertension, diabetes mellitus, hyperlipidemia, smoking, and eGFR, which were known to be associated with arterial stiffness and aortic calcification.^[7,14]^ Variance inflation factor was quantified for the multicollinearity of variables. In multivariate regression analysis, we performed relative weight analysis for which of these factors was most important in our regression model.^[24]^ To assess the relationship between age and aortic geometry, we used Pearson correlation method. A *P* value of < .05 was used to verify statistical significance. All statistical analyses were conducted using R version 3.4.1 (http://www.r-projec-t.org).

## 3 Results

### 3.1 Clinical characteristics of the study patients

The demographic and clinical characteristics of the study patients are shown in Table 1. The mean age was 65.4 ± 10.4 years, with 108 men (59.7%). The mean BMI was 24.2 ± 3.4 kg/m^2^. The prevalence of coronary artery disease, heart failure, stroke, and chronic kidney disease were 65.2%, 27.1%, 8.3%, and 39.2% respectively. Hypertension, diabetes, and hyperlipidemia were noted in 127 (70.2%), 64 (35.4%), and 80 (44.2%) patients, respectively. Seventy-one patients (39.2%) were smokers. Major laboratory findings did not deviate from their normal ranges. Renin-angiotensin system blockers, β-blockers, calcium channel blockers, and statins were already prescribed for 47 (26%), 95 (52.5%), 103 (56.9%), and 133 (73.5%) patients, respectively.

**Table 1:**
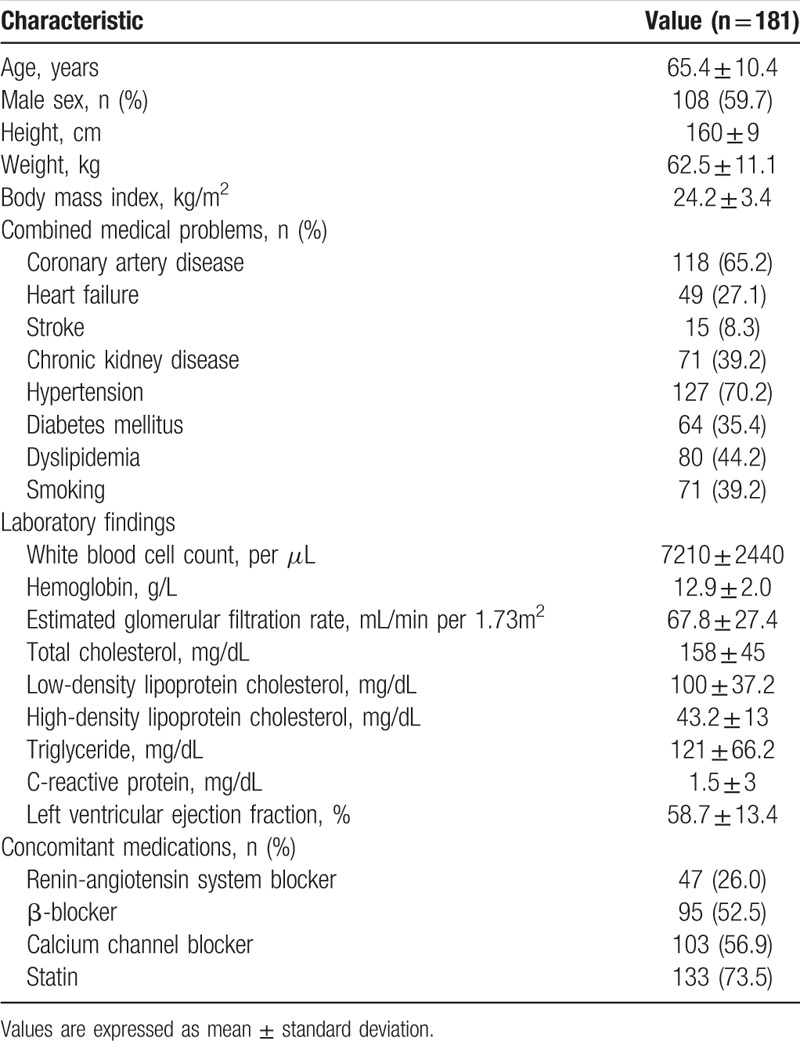
Clinical characteristics of the study patients.

Table 2 demonstrates the results of baPWV, aortic calcification, and aortic tortuosity measurements. The mean value of average baPWV was 1698 ± 435 cm/s and median value was 1643 cm/s (interquartile range [IQR], 1407–1916 cm/s). The mean value of aortic calcification, log of aortic calcification, and aortic tortuosity were 6,990 ± 9,695, 3.3 ± 0.9, and 1.9 ± 0.2, respectively, and median values were 3,360 (IQR, 1,083–9,424), 3.5 (IQR, 3.0–4.0), and 1.8 (IQR, 1.7–2.0), respectively.

**Table 2:**
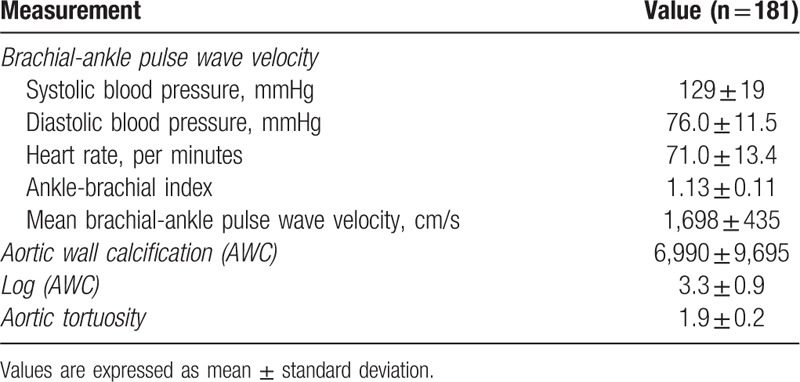
Pulse wave velocity and aortic geometry measurements.

### 3.2 Associations of clinical factors and baPWV with aortic calcification and tortuosity

**Figure 3:**
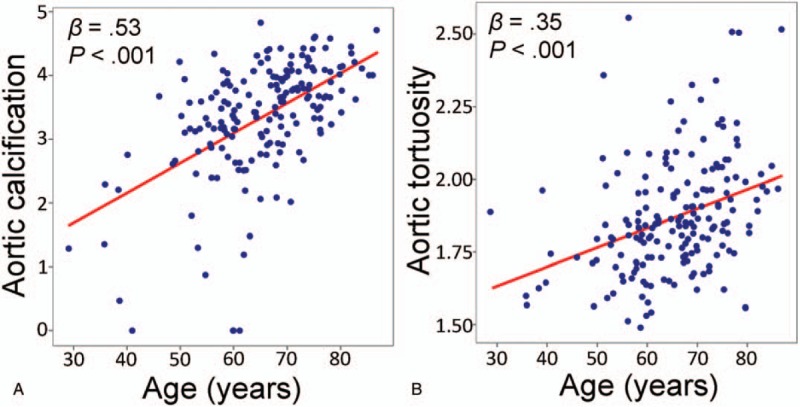
(A) Aortic calcification and (B) aortic tortuosity increased with age.

**Figure 4:**
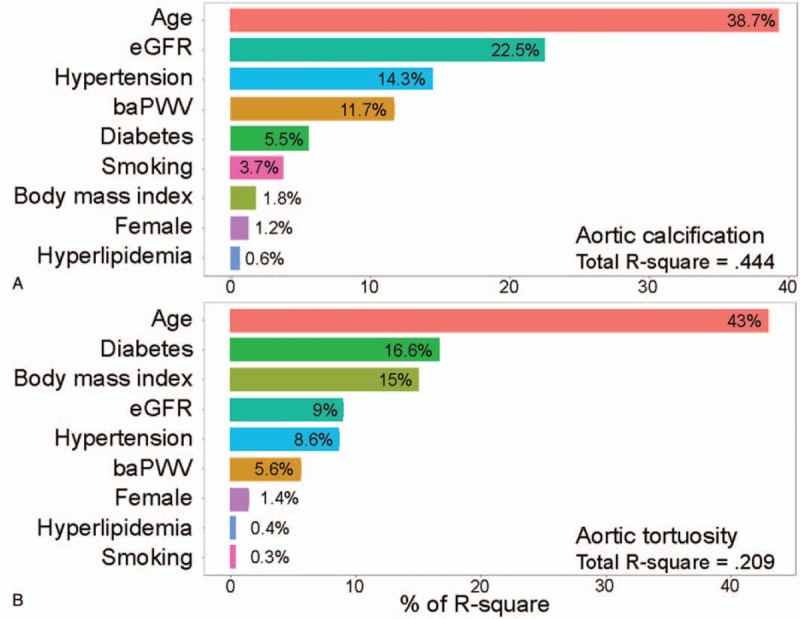
Relative importance for (A) aortic calcification and (B) aortic tortuosity. baPWV = brachial-ankle pulse wave velocity, eGFR = estimated glomerular filtration rate.

**Figure 5:**
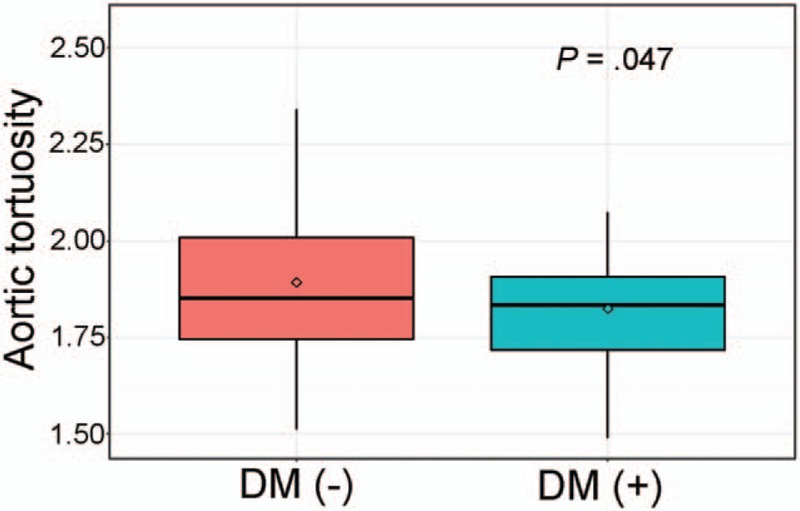
Comparison of aortic tortuosity between patients with and without diabetes mellitus. DM = diabetes mellitus.

**Figure 6:**
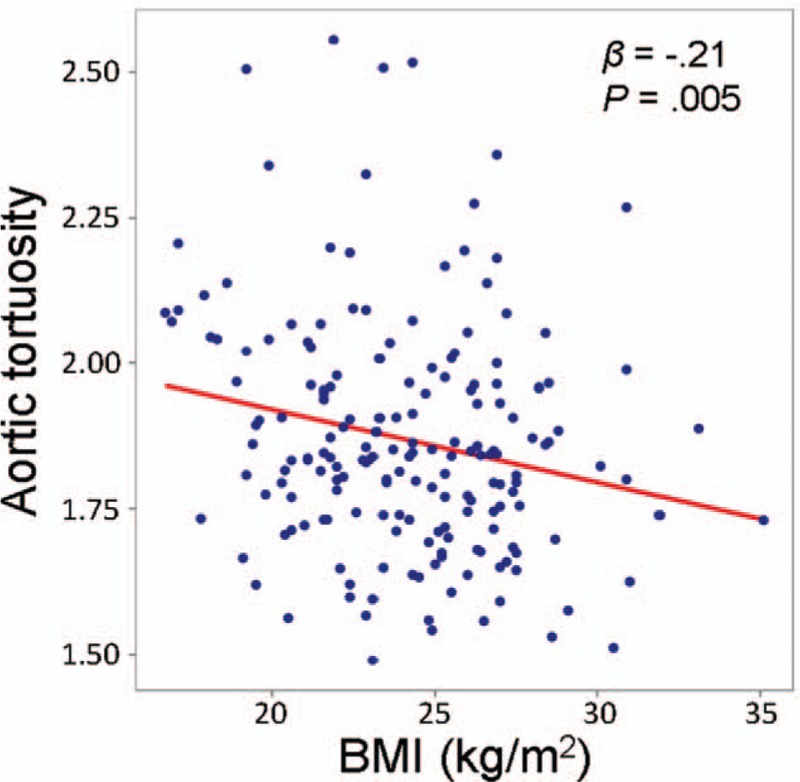
Linear correlation between BMI and aortic tortuosity. BMI = body mass index.

Table 3 shows the results of simple and multiple linear regression analyses of factors associated with aortic calcification. In simple linear regression analyses, aortic calcification was associated with age, hypertension, diabetes mellitus, eGFR, and baPWV. However, multiple linear regression analysis demonstrates that independent factors associated with aortic calcification were age (*β* = 0.37, *P* < .001), hypertension (*β* = 0.19, *P* = .003), diabetes mellitus (*β* = 0.12, *P* = .045), smoking (*β* = 0.17, *P* = .016), and eGFR (*β* = –0.25, *P* = .002). In multivariate analysis, baPWV showed no statistically significant correlation with aortic calcification (*P* *=* .123). Age, BMI, diabetes mellitus, eGFR, and baPWV showed a significant correlation with aortic tortuosity in simple linear regression analyses (Table 4). However, age (*β* = 0.34, *P* < .001), BMI (*β* = –0.19, *P* = .018), and diabetes mellitus (*β* = –0.21, *P* = .003) showed a significant association with aortic tortuosity in multiple linear regression analysis. The correlation between baPWV and aortic tortuosity was not observed in multiple linear regression analyses (*P* = .518). The correlation of age with aortic calcification (*β* = 0.53, *P* *<* .001) and tortuosity (*β* = 0.35, *P* < .001) is shown using a scatter plot in Figure 3. Age was the most important factor associated with both aortic calcification and tortuosity in relative importance analyses (Fig. 4). The eGFR and hypertension were the second and third most common factors associated with aortic calcification, while diabetes mellitus and BMI were those with aortic tortuosity. The value of aortic tortuosity was significantly low in patients with diabetes mellitus than in those without (1.8 ± 0.2 versus 1.9 ± 0.2, *P* = .019) (Fig. 5). A negative linear correlation between aortic tortuosity and BMI is demonstrated in Figure 6.

**Table 3:**
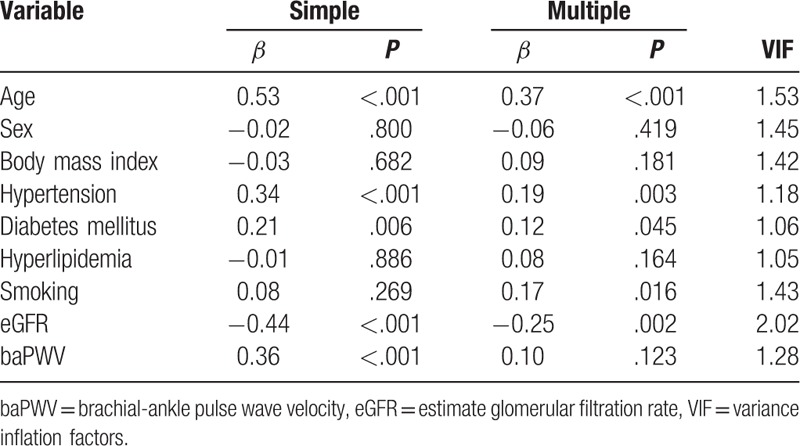
Simple and multiple linear regression analyses showing factors associated with aortic calcification.

**Table 4:**
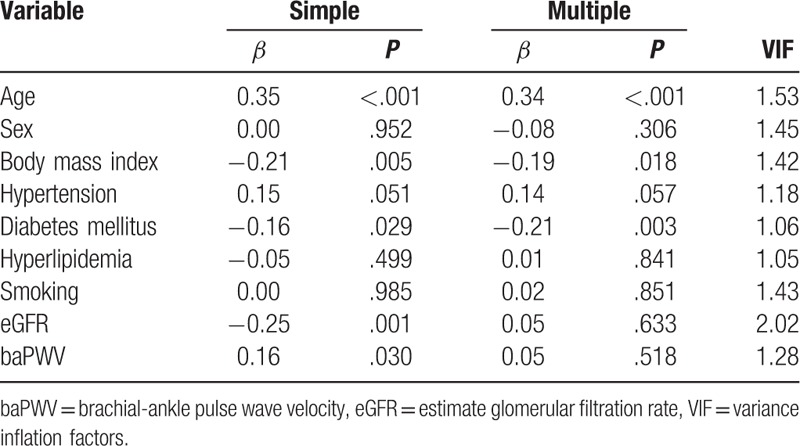
Simple and multiple linear regression analyses showing factors associated with aortic tortuosity.

## 4 Discussion

Our study aimed to investigate whether noninvasively measured arterial stiffness can predict aortic calcification and/or tortuosity. Aging, hypertension, diabetes mellitus, smoking, and renal impairment were associated with increased aortic calcification, while aging, low BMI, and non-diabetes were associated with increased aortic tortuosity even after controlling for potential confounders. Increased baPWV was associated with both increased aortic calcification and tortuosity in univariate analyses; however, their associations disappeared in multivariate analyses. Aging was the most powerful factor affecting both aortic calcification and tortuosity in our analyses. To the best of our knowledge, this is the first report investigating the association between baPWV and aortic calcification/tortuosity, although the results were negative.

Several studies have shown that arterial stiffness is associated with aortic calcification. Guo et al^[21]^ showed in that middle-aged men, annual changes in aortic calcification were significantly associated with arterial stiffness progression and incidence of aortic calcification during the follow-up period among patients without it at baseline. However, this result was derived from healthy men (hypertension 23%, diabetes 7%), and they made groups of aortic calcium scores, although the cutoff values are unknown. Shih et al^[25]^ demonstrated a significant association between aortic arch calcification measured by chest X-ray and baPWV in hemodialysis patients. However, they did not include hypertension or smoking as covariates, and chest X-ray is not a reliable modality for the evaluation of aortic calcification. Contrary to these results, there was no significant association between baPWV and aortic calcification in our study. Other traditional cardiovascular risk factors, such as aging, hypertension, diabetes mellitus, smoking, and lower eGFR, showed more strong associations with aortic calcification. These discrepant results among studies were due mainly to different study populations. Our results were obtained from patients with high cardiovascular risk profiles undergoing both CTA and baPWV. In addition, there could be the possibility that our data did not reach statistical significance in the association between baPWV and aortic calcification due to the relatively small number of study patients.

Our results showed that aging, hypertension, diabetes mellitus, smoking, and renal impairment were associated with increased aortic calcification. Aging and hypertension may put long-term mechanical and chemical stresses on the vessel wall, contribute to its structural changes.^[2]^ Diabetes can aggravate vascular calcification through the mechanism of vascular inflammation with vascular smooth muscle cells.^[26]^ Smoking, an important contributor to atherosclerosis, can affect vessel inflammation and worsen vascular calcification.^[27]^ An abnormal balance between calcium and phosphorous induced by renal impairment can cause vascular calcification.^[28]^

Aging was the most important contributor to increasing aortic tortuosity in this study. Cuomo et al^[29]^ reported the association of age-related changes in regional wall properties and geometry with computational modeling of the human aorta. They showed that tortuosity was increased with age by comparing the 30-year-old baseline model with the 40-, 60-, 75-year-old models. Continuous stresses on elastin fibers induce fragmentation and reduction in the vessel wall, and aging makes the aorta more tortuous through a process known as arteriosclerosis.^[30]^

There was a negative correlation between BMI and aortic tortuosity in our study. Although it is unclear whether obesity is related to vascular remodeling, arterial enlargement with elevated BMI was observed in several studies.^[31,32,33]^ It has been suggested that increased aortic tortuosity may be an adaptive mechanism for increased body mass.^[32]^ However, these studies were carried out using peripheral arteries, such as the carotid and brachial arteries, as well as vascular function tests, such as arterial stiffness, showing different results.^[31,32,33]^ Golledge et al^[34]^ demonstrated that obesity is independently associated with abdominal aortic aneurysm. They screened 12,203 men for abdominal aortic aneurysm using ultrasound, of whom 875 were diagnosed with abdominal aortic aneurysm. BMI had no association with abdominal aortic aneurysm, but waist circumference and waist-to-hip ratio had significantly positive odds ratios. Obesity may correlate with aortic geometry, but this needs to be further studied.

Our results showing the association between diabetes mellitus and aortic tortuosity are interesting. Similar findings were reported by Kamenskiy et al^[35]^ They analyzed 122 subjects using 3D reconstructions of CTA and showed that the presence of diabetes was associated with an unfold aorta, which may have been attributed to aging.^[19]^ Pathophysiological mechanisms underlying the association between diabetes and aortic tortuosity is still unknown. However, arterial tortuosity syndrome, characterized by extensive arterial elongation and torsion, as well as aneurysms of large and medium arteries, could be a clue to the mechanism. In patients with this rare autosomal recessive connective tissue disease, *SLC2A10* mutations were identified. This gene encodes the GLUT10 glucose transporter and has previously been proposed as a candidate gene for type 2 diabetes.^[36]^ Another study with mice carrying GLUT10 mutations showed abnormal elastogenesis with early elastic fiber proliferation of the arterial system,^[37]^ suggesting that diabetes could affect arterial tortuosity and might be some explanation of our study results. Further studies are needed to confirm our findings and hypothesis.

Besides its retrospective study design, this study has several limitations. First, our study has a limitation due to its small sample size, and there might be the possibility that the association between baPWV and aortic calcification/tortuosity could not reach statistical significance. Second, baPWV and CT were not performed on the same day. Third, as our study only included patients with documented cardiovascular disease or at high risk, this result cannot be generalized to other groups of patients. Finally, there is currently no standard method for measuring aortic calcification and tortuosity.

Our results showed that arterial stiffness assessed by baPWV was not the main contributor to aortic calcification or tortuosity. Traditional cardiovascular risk factors, including aging, hypertension, diabetes mellitus, smoking, and renal impairment, were associated with increased aortic calcification, while aging, low BMI, and non-diabetes were associated with increased aortic tortuosity. Further studies with a larger sample size are needed to confirm our results.

## Author contributions

**Conceptualization:** Kwang Nam Jin, Hack-Lyoung Kim

**Data curation:** Inki Moon, Hyeon Jeong Suh

**Formal analysis:** Inki Moon, Hyeon Jeong Suh

**Project administration:** Kwang Nam Jin, Hack-Lyoung Kim, Sang-Hyun Kim

**Resources:** Hack-Lyoung Kim, Woo-Hyun Lim, Jae-Bin Seo, Sang-Hyun Kim, Joo-Hee Zo, Myung-A Kim

**Supervision:** Sang-Hyun Kim, Joo-Hee Zo, Myung-A Kim

**Validation:** Woo-Hyun Lim, Jae-Bin Seo

**Visualization:** Kwang Nam Jin, Inki Moon

**Writing – original draft**: Kwang Nam Jin, Inki Moon

**Writing – review & editing**: Hack-Lyoung Kim
